# Procalcitonin-guided antibiotic therapy in adult sepsis: A biochemical perspective from a retrospective cohort study

**DOI:** 10.5937/jomb0-59980

**Published:** 2026-01-28

**Authors:** Yi Zhang, Zhe Liu, Chen Ma, Wenjing Wang, Wei Chen

**Affiliations:** 1 The First Affiliated Hospital of Xi'an Jiaotong University, Department of Clinical Laboratory, Xi'an, China; 2 The First Affiliated Hospital of Xi'an Jiaotong University, Department of Surgical Intensive Care Unit, Xi'an, China

**Keywords:** procalcitonin, sepsis, biomarkers, antibiotic therapy, C-reactive protein, white blood cells, retrospective study, prokalcitonin, sepsa, biomarkeri, antibiotska terapija, C-reaktivni protein, leukociti, retrospektivna studija

## Abstract

**Background:**

Procalcitonin (PCT), a biomarker closely associated with bacterial infections, has emerged as a valuable tool in guiding antibiotic therapy. In sepsis management, it may help optimise antibiotic use and improve clinical outcomes. This retrospective cohort study aimed to evaluate the effectiveness of PCT-guided antibiotic therapy in adult sepsis patients, with a particular focus on biochemical responses.

**Methods:**

We retrospectively analysed medical records of 110 adult sepsis patients admitted between January 2019 and December 2023. Patients were allocated to either a standard antibiotic group (n = 53) or a PCT-guided antibiotic group (n = 57). Key variables included demographic data, treatment duration, infection control metrics, and white blood cell (WBC) count, C-reactive protein (CRP), and PCT levels, among others, before and after therapy.

**Results:**

Compared with the standard group, the PCT-guided group exhibited significantly greater reductions in WBC, CRP and PCT levels (P&lt; 0.05), shorter antibiotic duration, fewer secondary infections, and improved antibiotic de-escalation rates. A higher complete response rate (17.54% vs. 3.77%) was observed in the PCT-guided group. No significant difference was found in 28-day mortality.

**Conclusions:**

PCT-guided antibiotic therapy led to more favourable changes in key biochemical markers and clinical outcomes, supporting its role as a biomarker-driven approach to antibiotic optimisation in sepsis management.

## Abstract (sr)

**Uvod:**

Prokalcitonin (PCT), biomarker usko povezan sa bakterijskim infekcijama, se pokazao kao dragoceno sredstvo u vođenju antibiotske terapije. U lečenju sepse može doprineti optimizaciji primene antibiotika i poboljšanju kliničkih ishoda. Ova retrospektivna kohortna studija je imala za cilj da proceni efikasnost antibiotske terapije vođene PCT-om kod odraslih pacijenata sa sepsom, sa posebnim osvrtom na biohemijske odgovore.

**Metode:**

Retrospektivno su analizirani medicinski kartoni 110 odraslih pacijenata sa sepsom, primljenih u periodu od januara 2019. do decembra 2023. godine. Pacijenti su raspoređeni u grupu sa standardnom antibiotskom terapijom (n = 53) ili grupu sa PCT-vođenom terapijom (n = 57). Ključne varijable su obuhvatale demografske podatke, trajanje lečenja, parametre kontrole infekcije, kao i broj leukocita (WBC), nivo C-reaktivnog proteina (CRP) i vrednosti PCT-a pre i posle terapije.

**Rezultati:**

U poređenju sa standardnom grupom, grupa sa PCT vođenom terapijom je pokazala značajno veće smanjenje nivoa leukocita, CRP-a i PCT-a (P&lt; 0,05), kraće trajanje antibiotske terapije, manju učestalost sekundarnih infekcija i bolje stope deeskalacije antibiotika. Veća stopa potpunog odgovora je zabeležena u PCT-grupi (17,54% naspram 3,77%). Nije uočena značajna razlika u 28-dnevnom mortalitetu.

**Zaključak:**

Antibiotska terapija vođena PCT-om je dovela do povoljnijih promena u ključnim biohemijskim markerima i kliničkim ishodima, što potvrđuje njenu ulogu u biomarkerima zasnovanom pristupu optimizaciji antibiotske terapije u lečenju sepse.

## Introduction

Sepsis remains a significant health concern, contributing to agony and healthcare costs [1] [2]. Despite advances in critical care, sepsis continues to challenge healthcare systems worldwide, with an estimated incidence of 30 million cases annually and a mortality rate ranging from 20% to 30% [3] [4] [5]. Timely identification and the immediate start of suitable antimicrobial treatment are key to enhancing the prognosis of sepsis patients [6] [7] [8]. However, excessive and prolonged use of antibiotics can lead to adverse effects, antibiotic resistance, and increased healthcare utilisation [9]. Therefore, there is a critical need for precision medicine approaches to optimise antimicrobial therapy in sepsis patients.

Procalcitonin (PCL), a prohormone of calcitonin, has emerged as a promising biomarker for guiding antibiotic therapy in sepsis [10] [11]. In response to bacterial infections, PCL levels rise rapidly, reaching peak concentrations within 6 to 12 hours, and returning to baseline levels upon resolution of the infection [12] [13]. This dynamic behaviour makes PCL an attractive marker for differentiating bacterial infections from other causes of systemic inflammation, thereby aiding in the rational use of antibiotics [14] [15]. Several studies have investigated the utility of PCL-guided antibiotic therapy in various clinical settings, demonstrating the potential to reduce antibiotic exposure, duration of therapy, and healthcare costs without compromising patient outcomes [16] [17].

The rationale for conducting this study stems from the pressing need to optimise antibiotic use in sepsis, minimise the emergence of antimicrobial resistance, and improve patient care. Traditional approaches to antibiotic therapy often rely on empirical broad-spectrum coverage, which can result in unnecessary exposure to antibiotics and their associated risks [18] [19]. In contrast, a biomarker-guided approach, such as PCL, has the potential to facilitate targeted and judicious use of antibiotics, aligning with the principles of antimicrobial stewardship and individualised patient care [20] [21].

Furthermore, the economic implications of antibiotic overuse and misuse cannot be overlooked [22]. In addition to direct costs associated with medication and hospitalisation, inappropriate antibiotic use contributes to indirect costs related to prolonged hospital stays, increased risk of complications, and the need for additional healthcare interventions [23]. Therefore, identifying strategies to optimise antibiotic use, such as PCL-guided therapy, holds promise for reducing the economic burden of sepsis while maintaining high-quality patient care.

Although a large amount of evidence supports the use of PCL to guide antibiotic therapy, there remains a need for further validation and exploration of its effectiveness in specific patient populations like adults with sepsis. This retrospective cohort study aims to address this gap by evaluating the efficacy of PCL-guided antibiotic therapy in sepsis patients at a single centre.

## Materials and methods

### Study population

The present study conducted a retrospective analysis of the clinical data of 110 adult sepsis patients admitted to our hospital from January 2019 to December 2023. The patients were divided into two groups based on the treatment approach: the standard antibiotic treatment group (n = 53) and the PCL-guided antibiotic treatment group (n = 57). This study was ethically approved by the Hospital's Ethics Committee for retrospective use of anonymised patient data, with informed consent waived.

### Inclusion and exclusion criteria

Eligible participants were adults aged 18 years diagnosed with sepsis who met at least two systemic inflammatory response syndrome criteria, had received either PCT-guided or standard antibiotic therapy, and possessed complete medical records. Patients were excluded if they were younger than 18 years, had incomplete records or missing essential data, received both PCT-guided and standard therapy, had a history of immunosuppression or chronic corticosteroid use (including glucocorticoids, calcineurin inhibitors, or anti-proliferative/metabolic agents) [24] [25], or had known acute or chronic elevations of PCT due to non-infectious causes.

### Aetiology and antibiotic selection rationale

The primary sources of infection were lower respiratory tract and intra-abdominal infections, with cultures (blood and sputum) obtained before antibiotic initiation. Common isolates included Streptococcus pneumoniae, Klebsiella pneumoniae, and Escherichia coli, while some patients exhibited clinical or radiological signs suggestive of atypical pathogens such as Mycoplasma pneumoniae or Legionella pneumophila, which are often undetectable by standard cultures. Accordingly, all patients received a standardised empiric regimen consisting of a β-lactam agent (e.g., ceftriaxone or piperacillin-tazobactam) for broad Gram-positive/Gram-negative coverage, combined with azithromycin for atypical coverage, in line with international guidelines for severe community-acquired sepsis and pneumonia [26]. PCT levels on therapy duration guided the focus of this study versus a fixed course, rather than on initial regimen selection.

### Intervention

In the PCT-guided group, discontinuation of broad-spectrum antibiotics was determined by daily PCT levels, with therapy stopped when values declined by 80% from peak or fell below 0.5 ng/mL; azithromycin (Changzhou Pharmaceutical Co., LTD; SFDA approval number H20057523) was administered intravenously at 500 mg once daily for 5 days. In the standard therapy group, patients received the same background therapy but continued antibiotics for a fixed 7-day course, with discontinuation based solely on physician judgment and not on PCT values. In both groups, azithromycin was frequently included at the clinician's discretion, particularly in suspected pulmonary infections, consistent with local guidelines. However, its use was not a focus of comparison in this study.

### Timing of biomarker measurement

The timing of PCT measurement is defined as follows: Initial PCT (reported in Table 1) refers to the first PCT value obtained at the time of sepsis diagnosis in the emergency department or upon hospital admission. Pre-treatment PCT reported in Table 2 refers to the PCT value measured immediately before the initiation of the study-specific antibiotic stewardship protocol (PCT-guided or standard strategy). This measurement typically occurred within 3-6 hours after admission, following initial resuscitation and assessment.

**Table 1:** Demographic characteristics between the two groups. Initial PCT: measured at the time of sepsis diagnosis.

Parameter	Ordinary Antibiotic Group<br>(n = 53)	PCL-guided Antibiotic Group<br>(n=57)	t/χ^2^	P-value
Age (years)	55.72±6.85	57.09±7.21	1.020	0.31
Gender (M/F)	32/21	37/20	0.087	0.769
BMI (kg/m^2^)	25.43±3.62	26.18±3.91	1.047	0.298
Comorbidities (count)	2.35±0.98	2.17±1.05	0.897	0.371
APACHE II score	21.65±3.41	22.13±3.61	0.722	0.472
Initial PCT levels (ng/mL)	2.88±1.25	3.02±1.38	0.538	0.592
therapeutic environment			0.2	0.655
Intensive Care Unit	12 (22.64%)	15 (26.32%)		
General medical ward	41 (77.35%)	42 (73.68%)		

**Table 2:** Comparison of laboratory parameters before and after protocol-specific treatment.

Parameter		Ordinary Antibiotic<br>Group (n=53)	PCL-guided<br>Antibiotic Group	t	*P-value*
WBC count (x10^9^/L)	Before treatment	12.27±3.45	12.09±3.11	0.288	0.774
	After treatment	10.42±1.21	9.24±0.48	6.682	<0.001
CRP (mg/L)	Before treatment	127.67±0.89	121.45±25.67	1.827	0.073
	After treatment	94.78±20.36	86.79±15.42	88.925	<0.001
PCT levels (ng/mL)	Before antibiotic initiation	5.89±2.34	5.12±2.13	1.801	0.075
	After antibiotic initiation	2.35±0.89	2.03±0.43	2.387	0.02

### Data collection

This single-centre retrospective cohort study evaluated the effectiveness of PCT-guided antibiotic therapy in adult sepsis patients using data extracted from medical records. APACHE II scores and PCT levels were measured the day before treatment initiation, while routine blood tests were performed within three days after therapy completion. Treatment response was assessed at the end of antibiotic therapy through clinical, laboratory, and microbiological parameters, and categorised as complete response (resolution of symptoms, normalisation of leukocyte count, >80% reduction in CRP and PCT, and confirmed or presumed pathogen eradication), partial response (50-80% biomarker reduction with clinical improvement but without full normalisation), stable disease (no significant change), or progressive disease (worsening clinical or laboratory indicators, septic shock, or radiological progression). This response framework was adapted from established sepsis trial criteria [27]. Additional parameters, including APACHE II score, D-dimer, and platelet count, were used to classify therapeutic effect into four categories further: I, complete relief; II, partial relief; III, stable disease; and IV, progressive disease.

### Laboratory measurements

All laboratory analyses were conducted in the Department of Laboratory Medicine under standardised conditions in accordance with manufacturers' protocols and accredited quality assurance programs. Procalcitonin (PCT) was quantified using an electrochemiluminescence immunoassay (ECLIA) on the Cobas e601 analyser (Roche Diagnostics, Mannheim, Germany), with a functional sensitivity of 0.02 ng/mL; two levels of commercial controls (Roche PreciControl, Mannheim, Germany) were assayed daily to ensure reproducibility and accuracy. C-reactive protein (CRP) was determined by an immunoturbidimetric assay on the Hitachi 7600 automated analyser (Hitachi High-Technologies, Tokyo, Japan), with internal quality control performed twice daily using manufacturer-provided control sera. White blood cell (WBC) and platelet counts were obtained on a Sysmex XN-1000 haematology analyser (Sysmex Corporation, Kobe, Japan), which underwent daily calibration with Sysmex control materials to minimise analytical drift. D-dimer concentrations were assessed by latex-enhanced immunoturbidimetry on the STA-R Evolution coagulation analyser (Diagnostica Stago, Asnières, France), with parallel use of internal quality control samples to monitor precision. The Acute Physiology and Chronic Health Evaluation II (APACHE II) score was calculated at admission using routinely collected biochemical and physiological data, including arterial blood gases, electrolytes, renal and liver function tests, and haematological parameters, thereby integrating multiple aspects of clinical biochemistry into patient stratification. All assays were subject to both internal quality control procedures and participation in external proficiency testing schemes, ensuring compliance with international standards in medical biochemistry. This rigorous laboratory framework underscores the reliability of biomarker quantification and aligns the study with the journal's focus on biochemical and laboratory-based evaluation in clinical medicine.

### Statistical analysis

Data analysis was performed with SPSS software. The normality of continuous data was assessed using the Shapiro-Wilk test. Continuous data with a normal distribution were expressed as mean±standard deviation (SD) and compared using the independent samples T-test. Categorical data were summarised as frequencies and percentages (n, %). Differences between groups in these categorical variables were assessed using the Pearson chi-square test. This specific approach for proportional data ensures an accurate representation of their distribution and allows for valid inter-group comparisons. A two-tailed p-value of less than 0.05 was considered statistically significant for all tests.

## Results

### Demographic characteristics

In this single-centre retrospective study comparing the effectiveness of PCL-guided antibiotic therapy to standard antibiotic therapy in adult sepsis patients, demographic characteristics were analysed (Table 1). The ordinary antibiotic group and PCL-guided antibiotic group showed no significant differences in age (55.72±6.85 vs. 57.09±7.21 years, t=1.020, *P*=0.31), gender distribution (M/F: 32/21 vs. 37/20, χ^2^ = 0.087, *P*=0.769), BMI (25.43±3.62 vs. 26.18±3.91 kg/m^2^, t=1.047, *P*=0.298), comorbidities count (2.35±0.98 vs. 2.17±1.05, t=0.897, *P* = 0.371), APACHE II score (21.65±3.41 vs. 22.13±3.61, t=0.722, *P*=0.472), and initial PCL (PCT) levels (2.88±1.25 vs. 3.02±1.38 ng/mL, t=0.538, *P*=0.592). These findings suggest that the groups were well-matched in terms of demographic and clinical characteristics, providing a solid foundation for the comparison of treatment outcomes.

### Laboratory parameters

Before treatment, there were no significant differences in WBC, CRP and PCT levels between the two groups (*P*>0.05) (Table 2). The PCL-guided group exhibited a notable decrease in white blood cell (WBC) count (×10^9^/L) from before to after treatment compared to the ordinary antibiotic group, with a statistically significant t value of 6.682 (*P*<0.001). Additionally, C-reactive protein (CRP) levels (mg/L) decreased significantly more in the PCL-guided group compared to the ordinary antibiotic group, as indicated by a t value of 88.925 (*P*<0.001). Moreover, the PCL levels (ng/mL) displayed a substantial reduction in the PCL-guided group compared to the ordinary antibiotic group, with a statistically significant t value of 2.387 (*P*=0.02).

These findings indicated that the levels of biomarkers WBC count (×10^9^/L) and CRP (mg/L) were altered in parallel with PCL (ng/mL). PCL-guided Antibiotic Group may have better treatment outcomes.

### Duration of antibiotic therapy

The comparison of the duration of antibiotic therapy between the ordinary antibiotic group and the PCL-guided antibiotic group revealed significant differences in several key parameters (Table 3). The PCL-guided group demonstrated a significantly shorter total duration of antibiotic therapy compared to the ordinary antibiotic group (6.91 ±1.87 days vs. 8.37±1.92 days, t=4.039, *P*<0.001). Furthermore, the PCL-guided group displayed reduced durations of ICU stay (4.62±1.28 days vs. 5.18±1.35 days, t=2.262, *P* =0.026), hospital stay (11.43±2.55 days vs. 12.75±2.67 days, t=2.636,* P* =0.01), and mechanical ventilation (32.19±8.64 h vs. 36.42±9.75 h, t=2.398, *P* =0.018). Additionally, the PCL-guided group had a significantly greater number of antibiotic-free days compared to the ordinary antibiotic group (5.21±1.98 days vs. 3.83±1.62 days, t=4.015, *P*<0.001). These findings suggest that PCL-guided antibiotic therapy may lead to a reduced duration of antibiotic therapy and healthcare utilisation.

**Table 3:** Comparison of duration of antibiotic therapy between the two groups.

Parameter	Ordinary Antibiotic Group<br>(n=53)	PCL-guided Antibiotic<br>Group (n=57)	t	*P-value*
Total duration (days)	8.37±1.92	6.91±1.87	4.039	<0.001
ICU stay (days)	5.18±1.35	4.62±1.28	2.262	0.026
Hospital stay (days)	12.75±2.67	11.43±2.55	2.636	0.01
Mechanical ventilation (h)	36.42±9.75	32.19±8.64	2.398	0.018
Antibiotic-free days	3.83±1.62	5.21±1.98	4.015	

### Infection control and mortality

The comparison of infection control and mortality between the ordinary antibiotic group and the PCL-guided antibiotic group revealed significant differences in several key parameters (Table 4). The PCL-guided group displayed a significantly faster resolution of sepsis compared to the ordinary antibiotic group (42.17±11.35 hrs vs. 48.56±12.93 hrs, t=2.749, *P*=0.007). Moreover, the incidence of secondary infections was not significantly lower in the PCL-guided group compared to the ordinary antibiotic group (43.39% vs. 35.08%, χ^2^ = 2.941, *P*=0.233). There were no significant differences in 28-day mortality between the two groups (26.42% vs. 22.81%, χ^2^ = 1.375, *P*=0.503). Additionally, the PCL-guided group demonstrated significantly lower rates of antibiotic escalation (60.38% vs. 42.11%, χ^2^ = 7.071, *P*=0.029) and higher rates of antibiotic de-escalation (39.62% vs. 38.60%, χ^2^ = 6.233, *P*<0.001) compared to the ordinary antibiotic group. These findings suggest that PCL-guided antibiotic therapy may contribute to a faster resolution of sepsis, reduced secondary infection rates, and improved antibiotic management in adult sepsis patients.

**Table 4:** Comparison of infection control and mortality between the two groups.

Parameter	Ordinary Antibiotic Group<br>(n=53)	PCL-guided Antibiotic Group<br>(n = 57)	T/χ^2^	P-value
Sepsis resolution (hrs)	48.56±12.93	42.17±11.35	2.749	0.007
Secondary infections (%)	23 (43.39%)	20 (35,08%)	2.914	0.233
28-day mortality (%)	14 (26.42%)	13 (22.81)	1.375	0.503
Antibiotic escalation (%)	32 (60.38%)	24 (42.11)	7.071	0.029
Antibiotic de-escalation (%)	21 (39.62%)	22 (38.60)	6.233	<0.001

### Efficacy

The comparison of efficiency between the ordinary antibiotic group and the PCL-guided antibiotic group revealed significant differences in treatment response (Table 5). The PCL-guided group exhibited a significantly higher rate of complete response compared to the ordinary antibiotic group (17.54% vs. 3.77%, χ^2^ = 9.082, *P*=0.028). There were no significant differences in the rates of partial response (59.65% vs. 60.38%), stable disease (17.54% vs. 16.98%), and progressive disease (5.26% vs. 18.87%) between the two groups. These findings suggest that PCL-guided antibiotic therapy may result in a higher rate of complete treatment response in adult sepsis patients, indicating its potential for improved treatment efficacy.

**Table 5:** Comparison of efficacy between the two groups.

Parameter	Ordinary Antibiotic Group<br>(n = 53)	PCL-guided Antibiotic Group<br>(n = 57)	χ^2^	P-value
Complete response	2 (3.77%)	10 (17.54%)	9.082	0.028
Partial response	32 (60.38%)	34 (59.65%)		
Stable disease	9 (16.98%)	10 (17.54%)		
Progressive disease	10 (18.87%)	3 (5.26%)		

## Discussion

The effectiveness of PCL-guided antibiotic therapy in the management of adult sepsis has been a topic of increasing interest and scrutiny [28] [29]. This single-centre retrospective cohort study assessed the effects of PCL-guided antibiotic treatment on adult sepsis patients. The results offer evidence supporting the benefits of this approach for better patient outcomes and healthcare efficiency.

Our results demonstrate several key findings related to the effectiveness of PCL-guided antibiotic therapy in adult sepsis. Firstly, in terms of laboratory parameters, the study revealed significant differences between the two treatment groups. The PCL-guided group exhibited more favourable changes in key markers, including white blood cell count, C-reactive protein levels, and PCL levels. These findings suggest that PCL-guided antibiotic therapy may lead to more effective management of systemic inflammation and more accurate targeting of antibiotic therapy, potentially contributing to improved patient outcomes.

Furthermore, the duration of antibiotic therapy, a crucial aspect of sepsis management, was reduced in the PCL-guided group compared to the standard antibiotic group. Shorter durations of ICU stay, hospital stay, mechanical ventilation, and a higher number of antibiotic-free days in the PCL-guided group accompanied this reduction in the total duration of antibiotic therapy. These findings indicate that PCL-guided antibiotic therapy may contribute to a more rapid resolution of sepsis, reduced healthcare utilisation, and a decreased burden on healthcare resources.

Infection control and mortality outcomes also demonstrated important differences between the two treatment groups. The PCL-guided group exhibited a faster resolution of sepsis, a lower incidence of secondary infections, and more favourable rates of antibiotic escalation and de-escalation. While there were no significant differences in 28-day mortality between the groups, the other infection control parameters suggest that PCL-guided therapy may contribute to a more targeted and effective approach to antibiotic management, potentially reducing the risk of secondary infections and related complications.

The treatment response efficiency differed significantly between the two groups. The PCL-guided group demonstrated a higher rate of complete treatment response compared to the standard antibiotic group. This finding underscores the potential of PCL-guided antibiotic therapy to improve treatment efficacy and optimise patient outcomes in adult sepsis patients.

Our findings are consistent with previous research demonstrating the potential of PCL-guided antibiotic therapy to optimise antibiotic use, minimise the emergence of antimicrobial resistance, and improve patient care in the context of sepsis [30] [31] [32]. The ability of PCL to rapidly respond to bacterial infections and guide the initiation and discontinuation of antibiotic therapy based on dynamic changes in PCL levels makes it a valuable tool for precision medicine in sepsis management [33] [34]. These results consistently demonstrate that while PCT guidance does not significantly reduce all-cause 28-day mortality, it successfully achieves another critical goal: substantially reducing antibiotic exposure without compromising survival outcomes. Mortality in sepsis is a complex endpoint influenced by numerous factors beyond antibiotic duration alone, including the timing of source control, adequacy of initial resuscitation, and host immune response. Therefore, the lack of mortality difference is not unexpected. The paramount value of our PCT-guided strategy lies in its ability to achieve non-inferiority in this hard endpoint while demonstrating superiority in key process outcomes, as evidenced by the significantly reduced antibiotic duration, shorter hospital and ICU stays, and lower rates of secondary infections. This combination of findings powerfully underscores the clinical utility and safety of PCT guidance. It confirms that we can safely curtail unnecessary antibiotic use, thereby potentially reducing antibiotic-related adverse effects and contributing to the global effort against antimicrobial resistance, without jeopardising patient survival.

These findings have substantial relevance for sepsis treatment and antimicrobial use management. The potential for PCL-guided antibiotic therapy to reduce unnecessary antibiotic exposure, shorten the duration of therapy, and improve infection control while maintaining high-quality patient care aligns with the principles of antimicrobial stewardship and individualised patient management [35]. This is particularly relevant in the current healthcare landscape, where the prudent use of antibiotics is essential to mitigate the growing threat of antimicrobial resistance and minimise healthcare-associated complications.

It is worth noting that this study has several strengths, including its focus on a specific patient population (adult sepsis patients), the use of a well-defined treatment approach (PCL-guided antibiotic therapy), and the comprehensive assessment of various clinical outcomes. However, some limitations should be considered. The study's retrospective design and single-centre setting may lead to biases and restrict the applicability of the results. Future studies should use prospective, multicenter approaches to validate PCL-guided therapy for sepsis better.

### Limitations

Our study has several limitations. First, the specific antibiotic regimen, which included azithromycin as a component, was uniform across all patients. While this was based on our institutional protocol for suspected severe community-acquired infections at the time, it may not reflect the heterogeneity of pathogens causing sepsis. The generalizability of our findings to settings employing different first-line antibiotic choices may be limited. However, the primary focus of this study was not on the choice of antibiotic but on the utility of PCT to guide the duration of therapy, which we believe is a strategy applicable across various antibiotic regimens.

## Conclusion

In conclusion, our study provides valuable insights into the effectiveness of PCL-guided antibiotic therapy in adult sepsis patients. The significant differences observed in laboratory parameters, duration of antibiotic therapy, infection control, and treatment response support the potential of PCL-guided therapy to optimise the use of antibiotics, improve patient outcomes, and reduce healthcare utilisation. These findings underscore the importance of precision medicine approaches, such as PCL-guided antibiotic therapy, in the management of sepsis and highlight the need for further research to validate and expand upon these findings.

## Dodatak

### Ethics approval and consent to participate

This study was approved by the Ethics Committee of the First Affiliated Hospital of Xi'an Jiaotong University in accordance with regulatory and ethical guidelines about retrospective research studies (No. LLSBPJ-2023-405). Informed consent was not required for this study as it only involved anonymised patient data, ensuring no risk to patient care.

### Consent for publication

Not applicable.

### Availability of data and materials

All data generated or analysed in this study are included in the present manuscript.

### Funding

The study was supported by the Natural Science Basic Research Program of Shaanxi Province (Youth) (2023-JC-QN-0898).

### Acknowledgements

We express our appreciation to the staff in the First Affiliated Hospital of Xi'an Jiaotong University for their technical assistance.

### Conflict of interest statement

All the authors declare that they have no conflict of interest in this work.
